# Immune checkpoint inhibitors and cancer-related cognitive decline: a propensity score matched analysis in active chemotherapy patients

**DOI:** 10.3389/fimmu.2025.1540442

**Published:** 2025-03-07

**Authors:** Guangmin Jian, Jiling Zeng, Jun Lu, Weidong Wang, Yongluo Jiang, Tong Huang, Yu Si Niu, Zhoufang Chai, Xin Qi, Nianqi Liu, Youlong Wang, Cantong Liu, Jiacai Lin, Guanqing Zhong, Yiming Li, Pengfei Zhu, Zong-qing Zheng, Fadian Ding, Xinjia Wang, Weizhi Liu, Ao Zhang, Yifei Ma

**Affiliations:** ^1^ Clinical Laboratory, First Affiliated Hospital of Zhengzhou University, Zhengzhou, Henan, China; ^2^ Department of Nuclear Medicine, State Key Laboratory of Oncology in South China, Collaborative Innovation Center for Cancer Medicine, Sun Yat-sen University Cancer Center, Guangzhou, Guangdong, China; ^3^ Department of Hepatobiliary Surgery, the First Affiliated Hospital of Shantou University Medical College, Shantou, Guangdong, China; ^4^ Department of Orthopedics and Spine Surgery, Cancer Hospital of Shantou University Medical College, Shantou, Guangdong, China; ^5^ Department of General Surgery, General Hospital of XinJiang Military Command, No.359 YouHaoBeiLu, Urumqi, XinJiang, China; ^6^ Acute Communicable Disease Epidemiology Division, Dallas County Health and Human Services, Dallas, United States; ^7^ Department of gynaecology and obstetrics, Hospital of Maternal and Child Care, JiangShan, Zhejiang Province, China; ^8^ Department of Gastroenterology, Hainan Hospital of Chinese PLA General Hospital, Sanya, China; ^9^ Faculty of Psychology, Institute of Educational Science, Huazhong University of Science and Technology, Wuhan, Hubei, China; ^10^ Department of General Surgery, Hainan Hospital of People's Liberation Army General Hospital, Sanya, Hainan, China; ^11^ Department of Clinical Laboratory Medicine, Cancer Hospital of Shantou University Medical College, Shantou, Guangdong, China; ^12^ Department of Neurology, Hainan Hospital of People's Liberation Army General Hospital, Sanya, Hainan, China; ^13^ Department of Clinical Laboratory, State Key Laboratory of Oncology in South China, Collaborative Innovation Center for Cancer Medicine, Guangdong Key Laboratory of Nasopharyngeal Carcinoma Diagnosis and Therapy, Sun Yat-sen University Cancer Center, Guangzhou, Guangdong, China; ^14^ Department of Neurosurgery, Beijing Tiantan Hospital Capital Medical University, Beijing, China; ^15^ Department of Neurosurgery, Neurosurgery Research Institute, The First Affiliated Hospital, Fujian Medical University, Fuzhou, Fujian, China; ^16^ Department of Neurosurgery, Binhai Branch of National Regional Medical Center, The First Affiliated Hospital, Fujian Medical University, Fuzhou, Fujian, China; ^17^ Department of Hepatobiliary and Pancreatic Surgery, the First Affiliated Hospital of Fujian Medical University, Fuzhou, China; ^18^ Institute of Abdominal Surgery, the First Affiliated Hospital of Fujian Medical University, Fuzhou, China; ^19^ Fujian Provincial Key Laboratory of Precision Medicine for Cancer, the First Affiliated Hospital of Fujian Medical University, Fuzhou, China; ^20^ Department of Hepatobiliary and Pancreatic Surgery, National Regional Medical Center Binhai Campus of the First Affiliated Hospital, Fuiian Medical University, Fuzhou, China; ^21^ Department of Orthopedics and Spine Surgery, the Second Affiliated Hospital of Shantou University Medical College, Shantou, Guangdong, China; ^22^ Lab for Post-traumatic Stress Disorder, Faculty of Psychology and Mental Health, Naval Medical University, Shanghai, China; ^23^ The Emotion & Cognition Lab, Faculty of Psychology and Mental Health, Naval Medical University, Shanghai, China

**Keywords:** immune checkpoint inhibitors, cognitive dysfunction, chemotherapy, immune-related adverse events, long-term trajectory

## Abstract

**Background:**

We investigated whether 1-year trajectories of cancer-related cognitive decline (CRCD) would be different in patients with chemotherapy combined with immune checkpoint inhibitors (chemoICI group) as compared with chemotherapy alone (chemo group).

**Methods:**

Participants scheduled with or without ICI were prospectively recruited from three academic hospitals and followed up for 1 year in four sessions. Subjective and objective CRCD were measured by Perceived Cognitive Impairment (PCI) and Montreal Cognitive Assessment (MoCA), respectively. Primary endpoints were MoCA and PCI score changes and minimal clinically important difference (MCID), which was defined as threshold for meaningful impairment events. Propensity score matching (PSM) was performed for group comparison using logistic regression with covariates including age, cancer stage, and baseline cognitive scores. Linear mixed models adjusted for repeated measures.

**Results:**

Out of 1557 recruited patients PSM yielded 460 patient pairs (1:1). Mean PCI and MoCA scores of both groups reached MCID at 12-month session in both groups. In chemoICI, MoCA score changes were significantly lower in the 12-month session, and PCI score changes were lower in the 6, 9, and 12-month sessions than chemo (P<0.05). One-year meaningful impairment events risks were 0.44 and 0.56 in chemoICI, significantly higher than that of chemo (0.35 and 0.38, P<0.01). Significant differences were found in mean event-free survival time in patients with and without irAE in chemoICI subgroup analysis.

**Conclusions:**

Our findings suggest that combining chemotherapy with ICIs may exacerbate CRCD compared to chemotherapy alone. However, reliance on screening tools and self-reported measures limits definitive conclusions. Future studies incorporating comprehensive neuropsychological assessments are warranted. This study underscores the importance of using comprehensive cognitive assessments in future research to better understand the impact of ICIs on cognitive function.

## Introduction

Cancer-related cognitive decline (CRCD) refers to subtle, long-lasting changes in cognitive function observed in cancer patients undergoing active treatment (1, 2). While self-reported cognitive complaints range from 16% to 60% (1, 2), studies using objective cognitive assessments have identified measurable deficits in 20-40% of cancer patients receiving chemotherapy or immunotherapy (3–5). CRCD, also termed ‘chemobrain’ when associated with chemotherapy, manifests as multidomain deficits including memory, executive function, and processing speed (3, 4, 6, 7). The term “CRCD” is preferred over “chemobrain” as it encompasses cognitive changes resulting from multiple cancer therapies beyond chemotherapy, including immunotherapy, hormonal therapy, and targeted agents. Its pathophysiology involves neuroinflammation, oxidative stress, and immune dysregulation (8), though mechanisms specific to immunotherapy remain poorly characterized. The assessment of CRCD primarily relies on validated cognitive tests tailored for oncology populations. Commonly used objective measures include the Montreal Cognitive Assessment (MoCA) and the Hopkins Verbal Learning Test-Revised (HVLT-R), both of which are sensitive in detecting mild cognitive impairment in cancer patients (9). Additionally, computerized cognitive batteries such as CANTAB have been employed to assess processing speed, memory, and executive function in cancer-related cognitive impairment. Functional imaging modalities like fMRI and PET scans provide insights into treatment-induced neural changes, further corroborating cognitive test results. These methodologies have been specifically validated in cancer cohorts and mitigate limitations associated with self-reported cognitive complaints (4, 10, 11).

In addition to chemotherapy, one of the most common mechanisms of cognitive impairment is inflammation or immune-related damage to the central nervous system (12, 13). Immunotherapies, particularly immune checkpoint inhibitors (ICIs), may contribute to CRCD through neuroinflammation, endothelial dysfunction, and direct neuronal injury. Systemic cytokine release (e.g., IL-6, TNF-α, IFN-γ) can disrupt the blood-brain barrier (BBB), leading to neurotoxic effects (6). Additionally, ICIs may promote T-cell infiltration into the CNS, contributing to direct neuronal damage. Immune-related adverse events (irAEs), including encephalitis and neuroinflammation, have also been associated with cognitive decline in patients receiving ICIs (14, 15). CRCD was associated with immune dysfunction because CRCD was traditionally induced via neuroinflammation, direct neurotoxic injury, endothelial dysfunction, and hormonal changes, involving a series of biomarkers such as cytokines, neurotrophic factors, and proteins in neurons (16). In fact, ICIs have become one of the therapeutic cornerstones in solid cancers (17). ICIs overcome immunosuppression induced by cancer cells or normal tissue microenvironment to augment intratumoral or tissue cytotoxicity (18). In addition, ICIs act systematically to cause off-target, deranged autoimmunity in normal organs (19). IrAEs, with reported incidence of 15% to 90%, may have prolonged characteristics with subclinical course (19), especially in the central nervous system.

Studies hypothesized that ICIs alone could cause cognitive decline through unknown forms of subclinical encephalopathy by means of cytokine dysregulation or systemic T lymphocyte activation (6, 14, 15). The cognitive process that depends on the interplay of CNS neurons may be damaged irreversibly and sub-clinically, a process that could be overlooked by oncologists (12). Although no pivotal studies have yet specifically investigated whether ICIs can aggravate cognitive impairment during chemotherapy treatment, the combination of chemotherapy and ICIs has become increasingly common in clinical practice. ICIs are now a standard treatment option for several cancers, including lung cancer, melanoma, and gastrointestinal cancers, where they are often combined with chemotherapy to improve patient outcomes. As of 2019, approximately 43.6% of U.S. cancer patients were eligible for ICI therapy, with up to 12.5% responding to it. While this figure encompasses both monotherapy and combination treatments, it underscores the growing integration of ICIs into cancer care (20). Given the rising prevalence of this treatment combination, it is crucial to investigate its potential impact on cognitive function, especially as both therapies independently contribute to cognitive decline (17, 21).

As ICIs are becoming prevalent in combined chemo-immunotherapy regimen for cancer therapy, we aim to evaluate cognitive function decline in the context of active chemotherapy. In this study, we selected both objective and subjective cognitive assessments to provide a comprehensive evaluation of CRCD. The MoCA was chosen as an objective screening tool due to its sensitivity in detecting subtle cognitive impairments, particularly in patients undergoing cancer treatments. For subjective assessment, we used the Perceived Cognitive Impairment (PCI) scale, which is a validated tool within the Functional Assessment of Cancer Therapy-Cognitive (FACT-cog) questionnaire. This combination of objective and subjective measures aligns with current recommendations, which emphasize the use of both types of assessments for a more thorough evaluation of CRCD (4, 11). This study aimed to compare longitudinal trajectories of both subjective and objective cognitive decline between patients receiving chemotherapy alone versus combined chemoimmunotherapy, using propensity score matching to control for confounding variables.

## Methods

### Research setting and design

We performed a prospective multi-center cohort study in solid cancer patients scheduled to receive active treatment in medical oncology departments of three medical centers: First Affiliated Hospital of Zhengzhou University, Sun Yat-Sen Cancer Center, and Affiliated Cancer Hospital of Shantou University Medical College. Recruitment for the current study started in 2020.1 and ended in 2021.8 and all patients were consecutively recruited (See Participant Eligibility Criteria). The primary aim was to compare objective and subjective CRCD in patients receiving chemotherapy alone (chemo group) and ICI combined with chemotherapy (chemoICI group).

The research was approved by institutional review boards of the Affiliated Cancer Hospital of Shantou University Medical College (Approval No. ST-ZLY-2020-716). The procedure was performed according to the Helsinki Declaration. Included patients had given written informed consent before participation. Reporting adheres to the Strengthening the Reporting of Observational Studies in Epidemiology (STROBE) checklist for cohort studies (https://www.equator-network.org/reporting-guidelines/strobe/).

### Participant eligibility criteria

Inclusion criteria of the current study included 1) patients with stage I to IV solid cancers scheduled with standard dose of chemotherapy (chemo) or chemotherapy + ICI at the research sites during study period; 2) age > 30 years; 3) immunotherapy-naive before inclusion for both groups; 3) prognostic survival > 1 year; 4) normal liver function tests and kidney function tests at cancer diagnosis.

Exclusion criteria at recruitment were: 1) known history of CNS tumors, trauma, or metastasis; 2) history of neurodegenerative disease (e.g. Parkinson’s Disease); 3) history of or concurrent targeted therapies of tyrosine kinase inhibitors; 4) concurrent radiotherapy; 5) history of the psychiatric disease; 6) alcohol use disorder or current prescribed/unprescribed narcotic users; 7) brain radiotherapy history; 8) high-risk of metabolic encephalopathy during treatment as evaluated by oncologists.

Participants underwent detailed neurological exams. If any neurological deficits were identified, radiological procedures of MRI were ordered. If brain lesions, including metastasis or other tumors, were found and diagnosed, further follow-up of neurological exams would be excluded. Key exclusion criteria during follow-up included: 1) inter-group change of therapy regimens into chemo or chemoICI group; 2) treatment discontinuation of over 3 months; 3) any medical condition not negotiable to follow-up assessment, including stroke and prolonged drug intoxication; 4) brain infections or metabolic encephalopathy as diagnosed by oncologists.

### Follow-up and cognitive assessment

The baseline assessment was conducted after the treatment modality was determined by oncologists and before treatment began. The cognitive assessment consisted of subjective questionnaires and objective tests, which were measured by Perceived Cognitive Impairment (PCI, the cognitive impairment domain of FACT-cog) and Montreal Cognitive Assessment (MoCA, a screening cognitive test), respectively. The cognitive assessment in this study utilized screening tools rather than comprehensive neuropsychological testing. The MoCA was selected as an objective screening measure due to its sensitivity in detecting mild cognitive impairment, though it does not replace a full neuropsychological battery. While full neuropsychological batteries provide a more comprehensive assessment, they are often impractical in real-world oncology settings due to time and resource constraints. Recent studies have confirmed that MoCA is more effective in assessing executive function, attention, and memory, cognitive domains frequently affected in cancer patients (22, 23).

The PCI scale, a component of the FACT-cog, was included to capture subjective cognitive complaints, which are frequently reported in cancer patients and correlate with quality of life (24). This dual approach aligns with current recommendations for preliminary cognitive screening in oncology settings, providing an accessible method for tracking cognitive changes over time. Practice effects were mitigated by analyzing score change rather than absolute values, though residual learning effects cannot be fully excluded. Subjective measures were administered to participants at recruitment (baseline, time 0) and 4 sessions during follow-up with a 3-month interval: 3, 6, 9, and 12-month sessions during the medical check-up clinic of the research setting, during which time patients had cancer checkups. Objective assessment was carried out at recruitment (baseline, time 0) and 3, 6, and 12-month sessions. Follow-up was set conveniently with medical checkups to minimize dropouts. Patients were given 100 yuan as compensation if all sessions were completed. During each session, blood tests, questionnaires, and psychometric scales were drawn and reported otherwise. Final data were compared between comparable groups in 2022.9 utilizing propensity score matching (PSM). Measure instruments and matching protocols were shown in **Supplementary Methods**.

### Study endpoint and outcome

Cognitive variables included PCI scores (self-reported cognitive complaints) and MoCA scores (objective cognitive function). PCI assessed memory, attention, and executive function complaints, while MoCA measured global cognition, including memory, executive function, and visuospatial abilities. The analysis focused on score changes over time and the occurrence of meaningful cognitive impairment events, defined by the minimal clinically important difference (MCID) threshold. The primary endpoint was the MoCA and PCI score change from baseline scores. The 0.5*standard deviation (SD) of baseline MoCA and PCI scores after PSM was set as the threshold for MCID, based upon the distribution method of calculus (25). This approach provides a clinically interpretable threshold, and it was widely accepted for preliminary studies in oncology populations (25). Therefore, any score decrease from baseline at any follow-up session, of > MCID would be marked as a meaningful subjective impairment event (MSIE, for PCI) or a meaningful objective impairment event (MOIE, for MoCA). Thus, score change from baseline as well as proportions of MOIE/MSIE could be compared in the chemo versus chemoICI group, and the proportions included prevalence (all cases) and incidence (new cases). As an exploratory outcome, *post hoc* analysis was done in the chemoICI group to identify the independent association between any incident irAEs and MOIE/MSIE incidence. The irAE was diagnosed as solicited during follow-up according to the National Cancer Institute Common Terminology Criteria for Adverse Events (26).

### Exposure and bias control

The only exposure was ICI treatment in primary outcome analysis, with other information set as the confounding variables in PSM analysis or linear regression models (see **Supplementary Methods**). Specifically, the demographic variables included age, sex, body mass index values (BMI), illiteracy state, and socioeconomic status (SES). The primary confounders included in the analysis were age, sex, baseline cognitive scores, chemotherapy regimen, and comorbidities. Although education level, occupational status, and premorbid intelligence can influence cognitive function, they were not included due to incomplete data, prioritization of treatment-related factors, and potential recall bias in self-reported premorbid intelligence. Instead, baseline cognitive scores were used as a proxy for pre-existing cognitive function (4). Clinical variables included cancer stage, pathological diagnosis, chemotherapy regimens (platinum-based or others), history of chemotherapy, diabetes diagnosis, and Eastern Cooperative Oncology Group-Performance Scores (ECOG-PS). PCI and MoCA scores, depression status, and opioid use during follow-up were also recorded as baseline variables that might affect cognitive functions (27). To control for learning effects, score change rather than absolute scores were represented and compared in two groups. We assessed quality of life levels using Functional Assessment of Cancer Treatment-General (FACT-G) at baseline (28, 29).

### Sample size estimation and statistics

To estimate the sample size needed for statistical significance, we presumed a clinically relevant difference in score change of 4 points between the two groups, as reported in prior observation cohorts (30), and it was calculated > 272 patient pairs to detect such presumed difference, assuming drop-out rate of 20%, with a statistical power of 90% and type I error of 0.05 (31–33).

Propensity scores were calculated with multiple logistic regression and associated variables included all variables as adjusting confounders. In propensity matching, a nearest greedy algorithm was adopted to give head-to-head (1:1) matching between each patient in the two groups. Caliper width of 0.2 was adopted in score matching without replacement. Matching competence was evaluated with standardized mean difference (SMD), but traditional methods of paired tests were also represented for each baseline variable (34). An SMD > (√ ((n1 +n2)/n1*n2))*1.96 was defined as imbalanced matching (n1 = n2 refers to pre-matched sample sizes) (35).

Equivalent statistical tests of difference of score change difference in propensity-score matched samples were performed with Wilcoxon signed-rank tests. Equivalence tests of paired proportion difference, or MOIE/MSIE difference, were performed with McNemar tests. PCI or MoCA score change difference in subgroups with or without incident irAE was compared by linear regression models to adjust for all baseline variables. To calculate incidence rate, or new case rate, of MOIE/MSIE during follow-up, Kaplan-Meier survival curve was adopted to estimate mean event-free survival (EFS) time of comparable groups or subgroups. As for the consideration of sensitivity and specificity, the MoCA has demonstrated sensitivity (82%) and specificity (76%) in detecting mild cognitive impairment in cancer cohorts (22), though its ability to distinguish immune-mediated neurotoxicity remains unvalidated. Misclassification risks due to overlapping irAE symptoms (e.g., fatigue, depression) were mitigated by excluding patients with metabolic encephalopathy or infections during follow-up. Additionally, the PCI scale, while sensitive to patient-reported cognitive complaints, may be influenced by emotional states such as anxiety or depression, which are common in cancer patients undergoing treatment (36). To address this, we adjusted for depression status in our analyses and excluded patients with psychiatric diagnoses. Paired and independent log-rank tests were adopted to compare difference of EFS rate. the power remains over 90% to calculate difference of EFS rate of over 10% difference in both paired and independent log-rank test, assuming two-sided, 5% type I error. Multivariate EFS analysis using proportional hazards model assessed the hazard ratio (95% confidence interval, CI) of irAE that adjusted for all baseline variables, and the continuous variable were not categorized to preserve integrity. Sample sizes were calculated with PASS (version15.0.), and all statistical analysis was performed in R (version 4.0.5) software. Plots of prevalence were drawn with Graphpad Prism (version 8).

## Results

### Participant characteristics before and after matching

**Figure 1:**
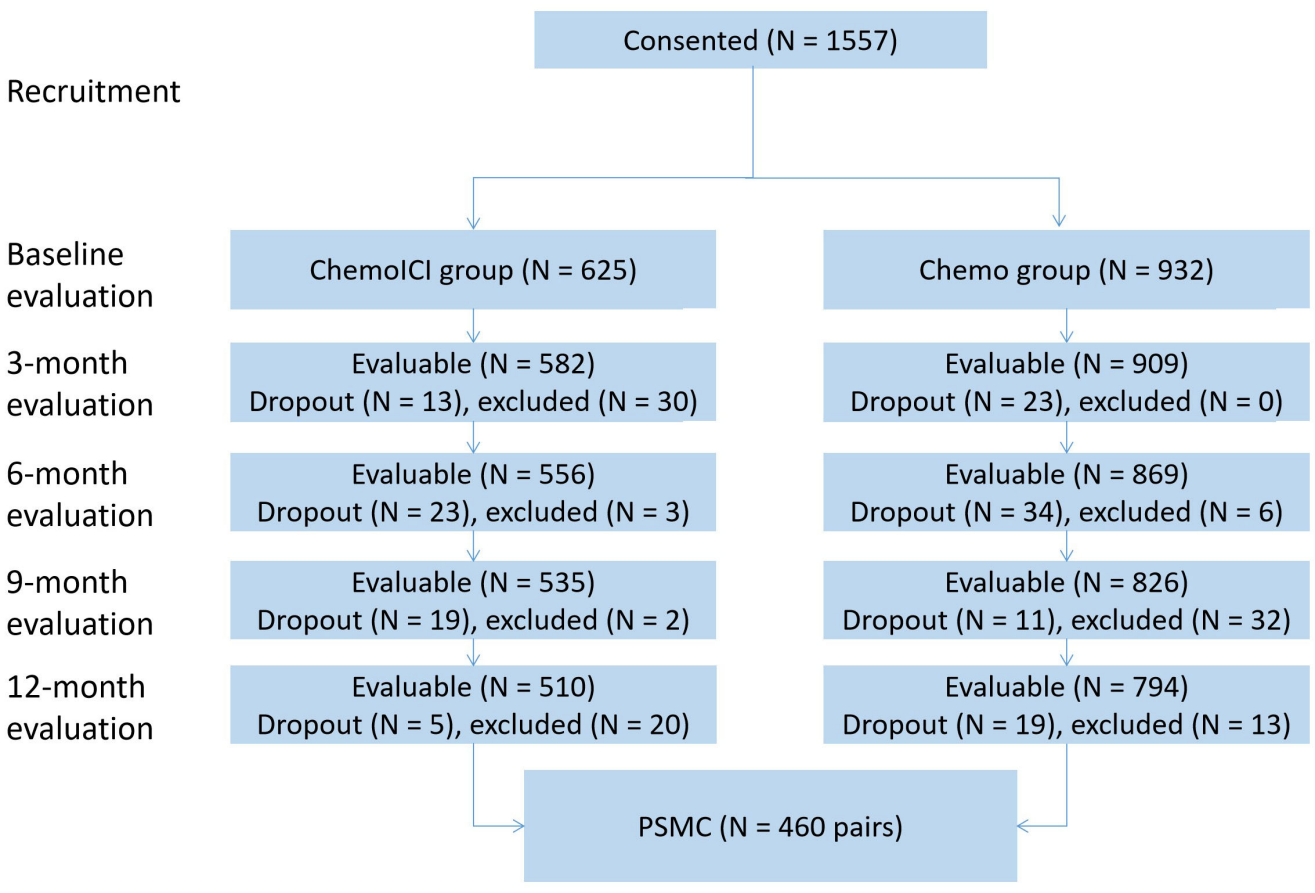
Recruitment flow diagram.

At baseline 1557 patients were enrolled and consented to the study protocol (**Figure 1**), reaching an initial sample size of 1304 (794 in the chemo group and 510 in the chemoICI group). All participants were from the Han ethnic group, and participants with illiteracy (n = 394, 30.2%) had significantly lower baseline PCI scores, but not MoCA scores, than non-illiterate participants. All baseline variables varied significantly between the chemo and chemoICI groups, except for sex, SES, and MoCA scores (**Table 1**).

**Table 1:** Baseline characteristics before and after propensity score matching.

Variables		Before matching		*p^a^*	After matching		SMD^c^	*p^b^*
		ChemoICI	Chemo		ChemoICI	Chemo		
Age	Mean (SD)	58.63 (12.22)	53.62 (14.41)	< 0.01	58.26 (12.22)	58.74 (12.47)	-0.04	0.67
	Distribution	53 (59 - 68)	54 (43 - 65)		59 (52 - 67)	59.5 (50 - 68)		
Sex	Male	324 (63.5%)	487 (61.3%)	0.42	294(63.9%)	288(62.6%)	0.03	0.73
	Female	186 (36.5%)	307 (38.7%)		166 (36.1%)	172 (37.4%)		
PCI score	Mean (SD)	47.69 (10.02)	44.95 (11.16)	< 0.01	47.28 (10.11)	47.41 (10.43)	-0.01	0.86
	Distribution	50 (43 - 55)	48 (37 - 54)		50 (43 - 55)	50 (41.25 - 55)		
MOCA score	Mean (SD)	21.51 (3.98)	21.65 (3.90)	0.53	21.6 (3.97)	21.47 (3.97)	0.04	0.62
	Distribution	22 (18 - 25)	22 (19 - 25)		22 (19 - 25)	22 (18 - 25)		
BDI score	Mean (SD)	7.43 (4.54)	8.30 (5.02)	< 0.01	7.49 (4.53)	7.61 (4.81)	-0.03	0.66
	Distribution	8 (3 - 11)	8 (4 - 12.75)		8 (4 - 11)	8 (3 - 12)		
Chemotherapy history^e^	Naive	466 (91.4%)	631 (79.5%)	< 0.01	416 (90.4%)	420 (91.3%)	0.03	0.71
	Yes	44 (8.6%)	163 (20.5%)		44 (9.6%)	40 (8.7%)		
Current chemotherapy	Pt-based	404 (79.2%)	562 (70.8%)	< 0.01	361 (78.5%)	362 (78.7%)	-0.01	1.00
	Others	106 (20.8%)	232 (29.2%)		99 (21.5%)	98 (21.3%)		
ECOG-PS	0 - 1	32 (6.3%)	89 (11.2%)	0.01	29 (6.3%)	35 (7.6%)	-0.01	0.93
	2	128 (25.1%)	214 (7.0%)		116 (25.2%)	116 (25.2%)		
	3	184 (36.1%)	245 (30.9%)		167 (36.3%)	146(31.7%)		
	4	166 (32.5%)	246 (31.0%)		148 (32.2%)	163 (35.4%)		
SES	Mean (SD)	12.00 (3.84)	11.88 (3.28)	0.55	8.13 (3.86)	8.22 (3.50)	0.02	0.83
	Distribution	8 (5 - 11)	8 (6 - 11)		8 (5 - 12)	8 (5 - 11)		
Stage^d^	II	126 (24.7%)	247 (31.1%)	0.04	119 (25.9%)	148 (32.2%)	0.07	0.31
	III	229 (44.9%)	324 (40.8%)		205 (44.6%)	170 (37.0%)		
	IV	155 (30.4%)	223 (28.1%)		136 (29.6%)	142 (30.9%)		
Cancer types	Head&Neck	151 (29.6%)	203 (25.6%)	< 0.01	134 (29.1%)	149 (32.4%)	-0.04	0.01
	Lung cancer	192 (37.6%)	215 (27.1%)		175 (38.0%)	132 (28.7%)		
	Gastrointestinal	167 (32.7%)	376 (47.4%)		151 (32.8%)	179 (38.9%)		
Opioid use^f^	Yes	178 (34.9%)	208 (26.2%)	< 0.01	146 (31.7%)	130 (28.3%)	< 0.01	0.25
	No	332 (65.1%)	586 (73.8%)		314 (68.3%)	330 (71.7%)		
BMI	Mean (SD)	22.81 (3.27)	23.69 (3.09)	< 0.01	23.04 (3.28)	23.05 (3.00)	0.07	0.95
	Distribution	22 (20 - 26)	24 (21 - 26)		23 (20 - 26)	23 (20.25 - 26)		
Diabetes	Yes	131 (25.7%)	164 (20.7%)	0.03	120 (26.1%)	118 (25.7%)	0.01	0.94
	No	379 (74.3%)	630 (79.3%)		340 (73.9%)	342 (74.3%)		

Categorical variables shown in numbers (%) and continuous varaibles in mean (SD) and median (1^st^ to 3^rd^ quartile).

*^a^* independent t test (continuous) or chi-square test (categorical);

^b^Wilcoxon signed rank test (continuous), McNemar non-parametric test (binary);

^c^SMD, standardized mean difference to show imbalance levels of variables after matching. Variable with SMD > 0.11 is considered poorly unmatched;

^d^cancer staging criteria, 2010 version, Union for International Cancer Control;

^e^over 2 months of chemotherapy (Pt-based, 28 patients); chemo-naive = new diagnosis (N = 429) and recurrent (N = 37);

^f^presciptions were recorded from 3months before recruitment to end of follow-up;

ECOG-PS, Eastern Cooperative Oncology Group-Performance Score; MOCA, Montreal cognitive assessment; Pt, platinum;BDI, Beck depression index; SES, socioeconomic status; PCI, perceived cognitive impairment; BMI, body mass index.

Propensity scores were calculated, and *post hoc* randomization was carried out to 1:1 match 460 pairs of patients from the chemo and chemoICI group, respectively, that dropped unmatched 50 (9.8%) and 334 (42.1%) patients from chemoICI and chemo group, respectively. Our a priori-defined imbalance threshold for any variable was 0.13 (see calculation formula in **Supplementary Methods**), and all variables had standardized mean difference (SMD) less than the threshold (**Table 1**). Non-parametric tests showed all variables were well balanced (*p* > 0.05) except for cancer diagnosis (*p* = 0.01). The results showed relatively satisfactory balancing of unmatched variables before PSM. After matching, both groups were similar in terms of baseline characteristics, especially PCI and MoCA scores.

Then, the threshold for MOIE and MSIE was calculated (0.5* SD of baseline scores). Our previously defined MCID of MoCA was -1.98 for both groups. MCID of PCI was -5.05 for the chemoICI group and -5.22 for the chemo group. Using this threshold, the statistical power of difference (< -5 or < -2) was calculated to be > 90% in the paired samples assuming a two-sided, type I error of 0.05. In addition, ICI types were relatively evenly distributed (**Supplementary Table 1**) in the matched chemoICI group.

Chemotherapy-only (chemo) group was over-sampled to allow for maximum matching for the chemotherapy plus immune checkpoint inhibitor (chemoICI) group. In total, 115 (18.4%) patients in the chemoICI group dropped or were excluded, and 138 (14.8%) patients dropped or were excluded from the chemo group, and the demographics of the excluded patients, including age and sex, did not differ from that of the included patients. All participants were of the Han ethnic group. The reasons for dropout and exclusion were in **Supplementary Materials.**

### Within-group and between-group comparison of MoCA and PCI score change

**Figure 2:**
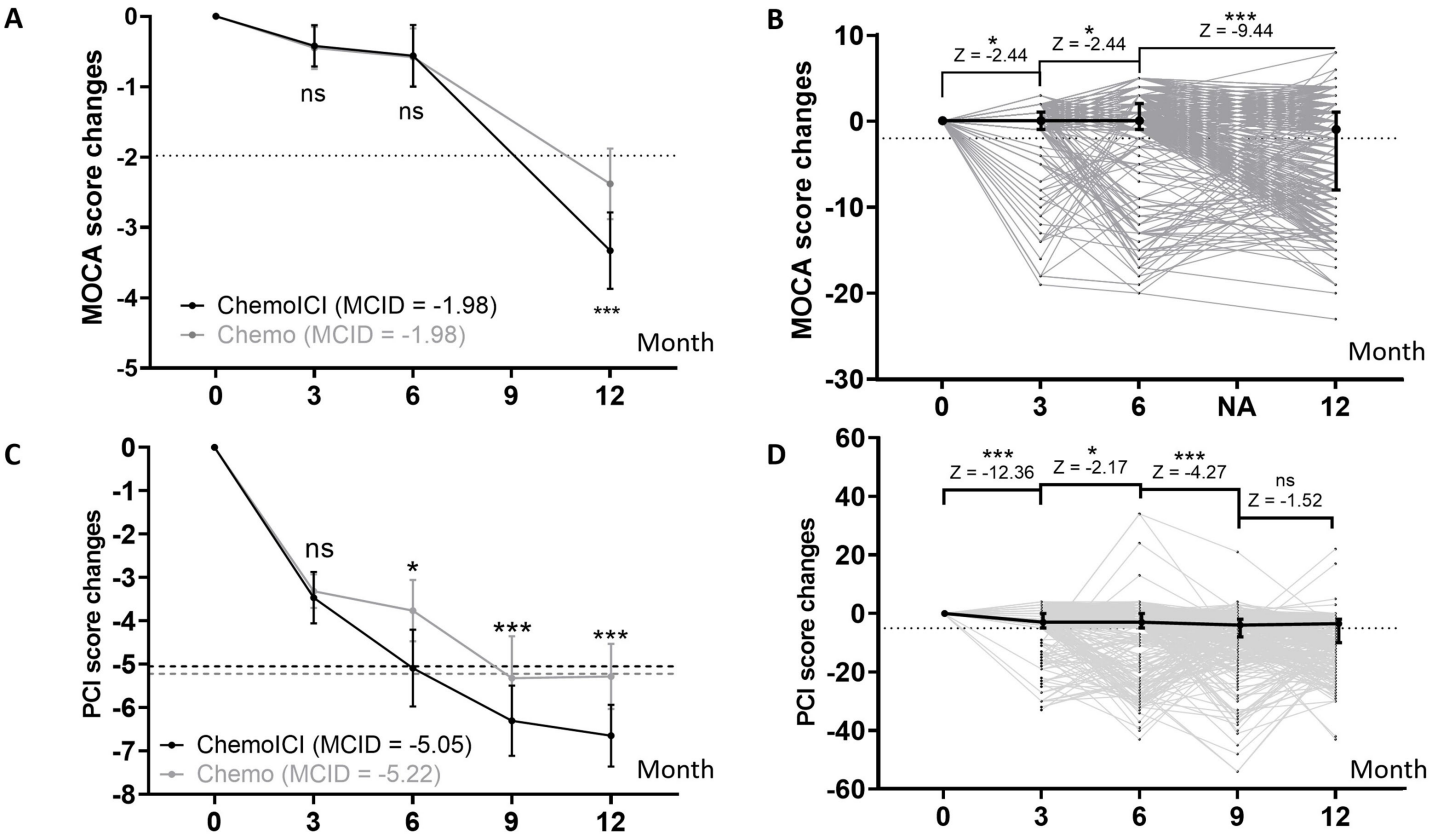
PCI and MoCA score change from baseline assessment. *, P<0.05; ***, P<0.001; ns, non-significant. **(A)** MoCA score changes in 3, 6, and 12-month sessions in the chemoICI group (black) and chemo group (gray) after propensity score matching (PSM), plotted as mean ± 95% CI (dot and error bar). Minimal clinically important difference (MCID) was plotted in each group. By the Wilcoxon signed-rank test, a significant difference of score change was found in the 12-month session (difference = -0.95, Z score = -2.69, p < 0.01), but not in the 3-month (difference = -0.03, Z score = -0.51, p = 0.96) or 6-month (difference = -0.02, Z score = -0.60, p = 0.55) session. **(B)** MoCA score change in chemoICI group, plotted as median ± inter-quartile range (dot and error bar). Wilcoxon signed rank test showed a significant difference of adjacent score change frombaseline to 12-month session (p < 0.05, p < 0.05, and p < 0.001). **(C)** PCI score change in 3, 6, 9, and 12-month sessions in the chemoICI group (black) and chemo group (gray), after PSM, plotted as mean ± 95% CI (dot and error bar). MCID was plotted in each group. Significant differences in score change were found between the two groups in the 6-month (difference = -1.32, Z score = -2.22, p < 0.05), 9-month (difference = -0.97, Z score = -6.0, p < 0.001), 12-month (difference = -1.45, Z score = -3.85, p < 0.001) session, but not in the 3-month (difference = -0.15, Z score = -0.92, p = 0.36) session, by Wilcoxon signed-rank test. **(D)** PCI score change in the chemoICI group, plotted as median ± inter-quartile range (dot and error bar). Wilcoxon signed rank test showed a significant difference of adjacent score change from baseline to 9-month (p < 0.001, p < 0.05, and p < 0.001) session but not in 12-month (p = 0.13) session.

MoCA score change were significantly different between chemoICI and chemo group in the 12-month session (*p* < 0.01), but not in the 3-month (*p* = 0.96) or 6-month (*p* = 0.55) session (**Figure 2A**). Within the chemoICI group, adjacent score change were all significantly different from baseline to 12 months (*p* < 0.05, *p* < 0.05, and *p* < 0.001), suggesting progressive decline (**Figure 2B**).

PCI score change were significantly different between the chemoICI and chemo group in the 6-month (*p* < 0.05), 9-month (*p* < 0.001), and 12-month (*p* < 0.001) sessions, but not in the 3-month (*p* = 0.36) session (**Figure 2C**). Within the chemoICI group, adjacent score change were significantly different from baseline to 9-month (*p* < 0.001, *p* < 0.05, and *p* < 0.001) session but not in the 12-month (*p* = 0.13) session (**Figure 2D**).

### Prevalence and incidence of MOIE and MSIE

**Figure 3:**
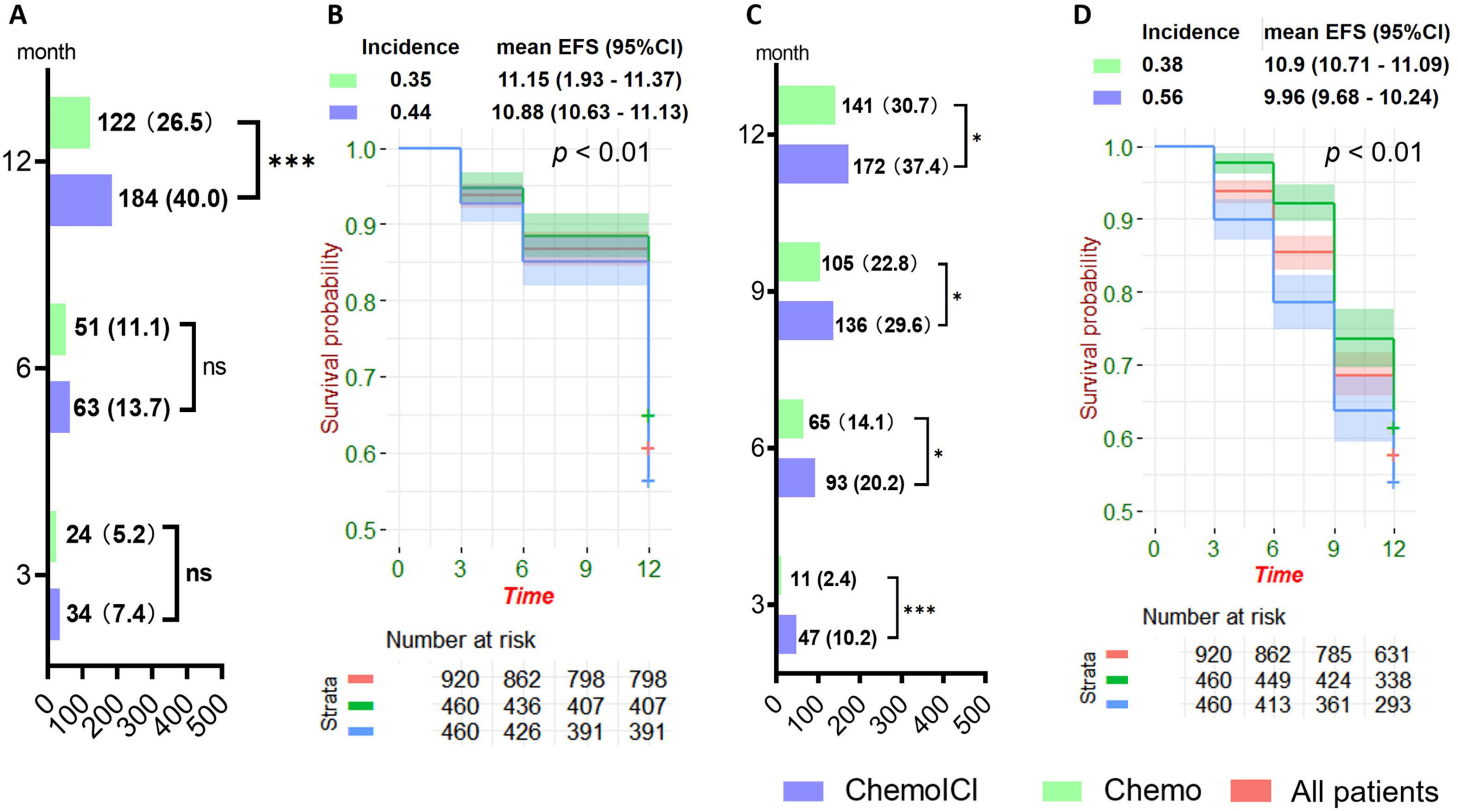
Prevalence and incidence of meaningful objective impairment event (MOIE) and meaningful subjective impairment event (MSIE). *, P<0.05; ***, P<0.001; ns, non-significant. **(A)** the prevalence of MOIE in chemoICI and chemo group in 3, 6, and 12-month sessions after matching, plotted as number (proportion). McNemar’s non-parametric test showed a significant difference in MOIE prevalence in 12-month (p < 0.001) but not in 3-month (p = 0.23) or 6-month (p = 0.27) sessions. **(B)** Kaplan-Meier survival curve of MOIE incidence (new case rate) in all patients (red), chemoICI (blue), and chemo (green) groups in 3, 6, and 12-month sessions after matching. Estimated mean eventfree survival (EFS) with a 95% confidence interval (95%CI) was shown and paired log-rank test showed a significant difference in EFS proportion (p < 0.01) in the 2 groups. **(C)** the prevalence of MSIE in chemoICI and chemo group in 3, 6, 9, and 12-month sessions after matching plotted as number (proportion). McNemar’s non-parametric test showed a significant difference in MSIE prevalence in all 4 sessions (p < 0.001, p = 0.02,p = 0.02, and p = 0.03 from 3 to 12-month sessions, respectively). **(D)** Kaplan-Meier survival curve of MSIE incidence in all patients (red), chemoICI (blue), and chemo (green) groups in 3, 6, 9, and 12- month sessions after matching. The paired log-rank test showed a significant difference in EFS proportion (p < 0.01) in the 2 groups.

Prevalence of MOIE was growing with time in both groups but was significantly different only in the 12-month session (40.0% in chemoICI vs. 26.5% in chemo, **Figure 3A**) during follow-up. The twelve-month incidence rate of MOIE was 0.35 (chemo) vs. 0.44 (chemoICI). Kaplan-Meier survival curve estimated EFS time of chemoICI to be 10.88 (95%CI 10.63 - 11.13) months, significantly shorter (*p* < 0.01) than EFS of chemo (mean = 11.15, 95%CI 10.93 - 11.37 months, **Figure 3B**).

The prevalence of MSIE was also growing with time in both groups and was significantly different in all sessions (**Figure 3C**). Kaplan-Meier survival curve estimated EFS time of chemo was estimated to be significantly shorter than chemoICI (*p* < 0.01, **Figure 3D**), with MSIE incidence rate of 0.56 in chemoICI and 0.38 in chemo group.

### Cognitive impairment in patients with and without irAEs: exploratory analysis

**Figure 4:**
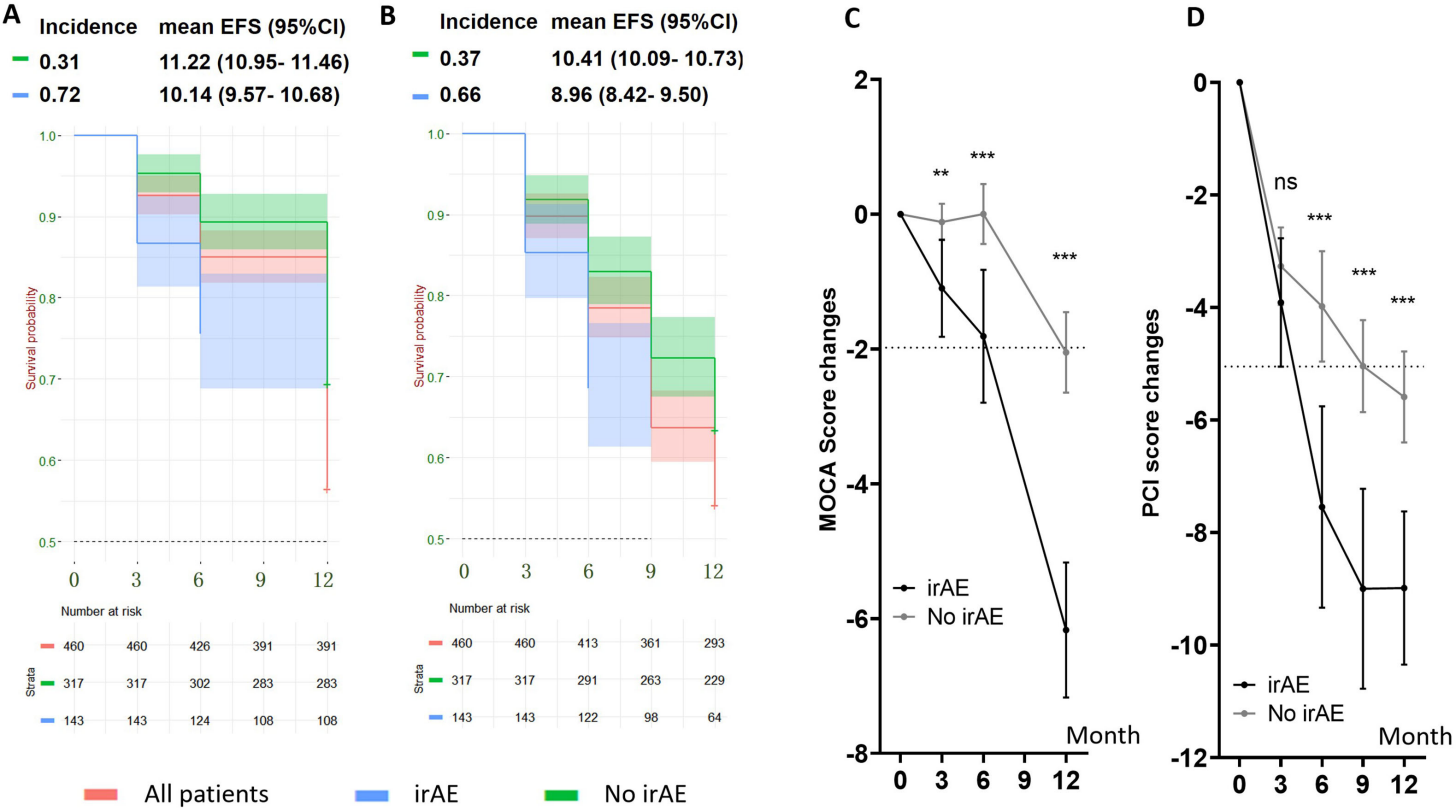
Cognitive impairment in subgroups with and without follow-up immune-related adverse events (irAEs) in the chemoICI group. **, P<0.01; ***, P<0.001; ns, non-significant. **(A, B)** Kaplan-Meier survival curve of MOIE incidence **(A)** and MSIE incidence **(B)** in patients with irAE (blue) and without irAE (green), with mean EFS survival and 95%CI shown. Independent log rank test showed a significant difference in EFS proportion in the two subgroups for both MSIE (p < 0.01) and MOIE (p < 0.01). **(C)** MoCA score change in patients with (black) and without (gray) irAE. Differences in decline were compared with linear regression analysis that adjusted for all baseline variables. Significant differences were found in 3-month (mean = 0.98, 95%CI = 0.36 - 1.61, p < 0.05), 6-month (mean = 1.81, 95%CI = 0.88 - 2.75, p < 0.001), and 12-month (mean = 4.12, 95%CI = 3.00 - 5.23, p < 0.001) session. **(D)** PCI score change in patients with (black) and without (gray) irAE. Significant differences were found in 6-month (mean = 3.56, 95% CI = 1.68 - 5.45, p < 0.001), 9-month (mean = 3.96, 95%CI = 2.26 - 5.66, p < 0.001) and 12-month (mean = 3.40, 95%CI = 1.89 - 4.90, p < 0.001) session, but not in the 3-month session (mean = 0.64, 95% CI = -0.64 -1.92, p = 0.54) after adjusting for baseline variables.

An exploratory, *post hoc* analysis was done to identify the independent relationship between irAE and cognitive impairment in the chemoICI group. Overall, 143 of 460 patients (31.1%) reported and were diagnosed with irAEs. After adjusting for all baseline variables, irAE was significantly associated with the 12-month incidence of MSIE and MOIE in the chemoICI group (*p* < 0.01 for both, **Supplementary Table 2**). The hazard ratio was 2.2 (95%CI 1.64 - 2.94) for MOIE and 2.11 (95%CI 1.59 - 2.8) for MSIE, and there was a significant difference of mean EFS time in patients with and without irAE (**Figures 4A, B**). Significantly higher MCOA and PCI score change were found in patients with irAEs (**Figures 4C, D**) that are adjusted for all baseline variables.

## Discussion

Our study investigated the severity, incidence, and prevalence of CRCD in patients receiving chemotherapy plus ICI and chemotherapy alone. We found both statistical and clinical differences between the two treatment groups in a 1-year follow-up study. To date, this may be the first report to identify the role of ICI in affecting cognitive function decline in chemotherapy-active cancer patients.

In our study, although earlier sessions during follow-up did not show a significant difference between chemo and chemoICI in MoCA score trajectories, the difference at the 12-month session suggested late-onset CRCD. Measurement using FACT-cog showed score change difference and prevalence in nearly all sessions. Indeed, discrepancies between objective and subjective screening measures may arise in the same domain and subjective measurement accuracy may affected by their physical and psychological status themselves at the time (36). Parallel design of objective and subjective measures precludes bias of expectancy or learning effects, and relatively large sample size ensured lower SD of mean score change (37). Another strength of subjective measurement includes greater sensitivity in finding subtle differences in CRCD (38). Nevertheless, since patients with chemotherapy may induce severe CRCD and the effects of ICI have been unknown so far, we adopted two measurements in this pilot investigation of ICI-related cognitive decline (39). MoCA and PCI were selected as co-primary cognitive measures to capture both objective and subjective cognitive changes.

While MoCA is widely used for screening mild cognitive impairment, it does not replace comprehensive neuropsychological testing. Comprehensive neuropsychological batteries, while ideal, are often impractical in clinical and real-world research settings due to time constraints and patient burden. Recent studies have recommended MoCA as a feasible and effective screening tool for detecting subtle cognitive impairments in chemotherapy- and immunotherapy-treated patients (22, 23). However, given the practical constraints of clinical oncology settings, MoCA provides a feasible alternative for detecting cognitive changes. We acknowledge its limitations and emphasize the need for further validation in the context of CRCD and immunotherapy. The PCI scale from FACT-cog was included to assess self-perceived cognitive difficulties, which are clinically relevant but often underrecognized (6). Patients’ subjective reports of cognitive impairment are generally more severe than objectively measured, and this pattern is often attributed to psychological factors such as anxiety, depression, fatigue, or insomnia, which have a greater impact on perceived cognitive problems than performance on objective tests (33). To mitigate bias, we excluded patients with psychiatric diagnoses, adjusted for depression status in analyses, and combined the subjective and objective assessments. The sensitivity and specificity of MoCA and PCI in capturing meaningful cognitive changes in the context of irAEs warrant further investigation. While MoCA is effective in detecting mild cognitive impairment, its ability to distinguish between chemotherapy-induced cognitive decline and immune-mediated neurotoxicity is not well established. Similarly, PCI, as a subjective measure, may be influenced by emotional factors such as anxiety or depression, which are common in patients experiencing irAEs. Future studies should consider integrating objective biomarkers (e.g., inflammatory cytokines, neuroimaging) to better characterize the underlying mechanisms of cognitive decline in patients receiving ICIs.

Our study contributes to the field by comparing chemo-ICI and chemotherapy-only groups over a 12-month period, using a real-world, longitudinal approach. These findings add to the growing evidence on ICI-related cognitive changes, addressing a gap in research on combination therapies (4, 11, 40). In the past decade, studies focused on chemotherapy as a main cause of CRCD in non-brain cancers although endocrine and targeted therapies were covered as well (30, 41). The chemo group as a control in current research reported similar findings, with the highest prevalence of 26.5% and a 1-year incidence of 35%. Recent studies suggest that ICIs can contribute to cognitive impairment through neuroinflammation and immune-related neurotoxicity. Increased cytokine activity (IL-6, TNF-α, IFN-γ) and T-cell infiltration may affect brain function, leading to cognitive decline. Neuroimaging data indicate changes in white matter integrity and functional connectivity after ICI treatment. While research on ICI-induced cognitive effects is still emerging, these findings highlight the need for further investigation, particularly in chemo-ICI combinations. The observed association between irAEs and CRCD supports the hypothesis that systemic immune activation may exacerbate neuroinflammation, potentially via cytokine-mediated blood-brain barrier disruption (42). Future studies should directly measure inflammatory markers (e.g., IL-6, TNF-α) to test this mechanism. In the chemoICI group, MOIE incidence reached 44%, 9% higher than that of the chemo group, and self-reported difference doubled in MSIE incidence (18%). Also, as within-group group comparison showed a significant decrease over time in chemoICI, profound and long-term persistence of CRCD could exist beyond 1-year follow-up, given the characteristic irreversibility of neurological irAEs.

Common checkpoint inhibitors include anti-CTLA-4 antibodies (e.g., ipilimumab), anti-PD-1/PD-L1 antibodies (e.g., pembrolizumab, nivolumab), et al. While the brain is the “immune privileged” organ in the body owing to the presence of the blood-brain barrier which prevents immune cells from penetrating the brain, the connection between the lymphatic system and the meningeal lymphatic system allows the crossing of T-cells and cytokines and could explain the effect of immunotherapy on central tumors (42). The mechanisms of neurological sequelae from systemic therapies are not completely understood, although some MRI and PET studies suggest structural and metabolic deficits and the ongoing cog-immune research aims to provide information for patients on the impact of immunotherapy on cognitive functions, and the evaluation of collection of blood samples and investigation of neurobiological mechanisms from brain slices will be conducted (43).

Strategies for managing CRCD include cognitive rehabilitation, physical exercise, and pharmacological interventions. Cognitive training programs have shown benefits in improving memory and executive function. Regular physical activity may help by reducing inflammation and promoting neuroplasticity. Pharmacological options, such as modafinil, methylphenidate, and melatonin, have been investigated for cognitive and fatigue-related symptoms, though further research is needed to confirm their efficacy, particularly in chemo-ICI-treated patients (11). The results of this study support seeking remission of CRCD from an immunologic perspective in the future.

Strengths of this study included balanced baseline variables through PSM pairs, multicenter design, relatively large sample, and longitudinal follow-up with relatively good adherence, but there are limitations to be discussed. First of all, MoCA is regarded as a screening tool for mild cognitive impairment and lacks specificity in finding severe CRCD (44), although a study of 15 patients with ICI treatment proved its feasibility (45). Indeed, although a standard cognitive test battery can precisely assess the level and sub-domain of CRCD in optimal settings (46), the test generally requires patient adherence and professional consultation in large-sample, multicenter settings (15). Second, allocation into chemoICI or ICI was based upon oncologists’ decisions rather than randomization. Potential bias in real-world settings could exist, and PSM was done in our study as a salvage protocol. While we aimed to account for key confounders, education level, occupational status, and premorbid intelligence were not included in the analysis due to inconsistent data availability, prioritization of treatment-related confounders in PSM, and concerns about selection bias in self-reported premorbid intelligence measures. Instead, baseline cognitive scores were used as a proxy for pre-existing cognitive function, though this approach has limitations. Third, although PSM balanced most covariates, residual imbalance in cancer diagnoses (p = 0.01) persisted, likely reflecting the preferential use of ICIs in specific cancer types (e.g., lung cancer) in clinical practice. This may limit the generalizability of our findings to cancers where ICIs are less commonly used. Future studies should prioritize cancer-specific cohorts to further validate these results.

## Conclusions

This prospective cohort study provides an initial exploration of the cognitive effects of chemotherapy combined with ICIs, demonstrating a potential association with greater cognitive decline, particularly in patients experiencing irAEs. These findings contribute to the growing body of evidence on CRCD and highlight the importance of monitoring cognitive outcomes in patients receiving immunotherapy. However, as this study relies on screening tools, further research using comprehensive neuropsychological assessments and neuroimaging is needed to confirm these findings, refine mechanistic understanding, and guide clinical interventions.

## Data Availability

The original contributions presented in the study are included in the article/**Supplementary Material**. Further inquiries can be directed to the corresponding authors.
