# Supplementary material for: Immune checkpoint inhibitors and cancer-related cognitive decline: a propensity score matched analysis in active chemotherapy patients
Source: Front Immunol. 2025 Mar 7;16:1540442. doi: 10.3389/fimmu.2025.1540442 (PMC11925948; doi:10.3389/fimmu.2025.1540442)
Supplement: Supplementary file 1 [file DataSheet1.docx]

**Supplementary methods**

**Measurement**

Subjective cognitive impairment was measured by perceived cognitive impairment (PCI, 20 items), which is one of the domains in the functional assessment of cancer treatment-cognitive (FACT-cog) for cognitive ability involving other 3 domains: perceived cognitive abilities (9 items), comments from others (4 items), and impact on quality of life (4 items)^1-3^. Because this study focused on cognitive impairment, only the PCI domain was used. Likert scaling method was applied in scoring each question (0 to 4) and lower scores suggested greater subjective cognitive decline in patients receiving chemotherapy and endocrine therapy^4,5^.

Objective measurement was performed with Montreal Cognitive Assessment (MOCA, Chinese version 7, http://www.mocatest.org). MOCA is a brief instrument to globally screen cognitive impairment in domains including naming, visuospatial, language, attention, abstraction, orientation, and delayed recall, totaling 30 points. MOCA has been validated in testing ICI-related cognitive decline in a small sample of cancer patients previously as a feasibility study^6^ and in samples of non-cancer subjects^7,8^. For patients with partial or complete illiteracy, we administered the basic form of MOCA (MOCA-B) and standardized the score into 30 points. Interviewers of residents who speak dialects interviewed local patients to avoid misunderstanding. Patients with hearing or visual disabilities were assisted by third-party translators. Interviewers were trained in psychometrics at Huazhong University of Science and Technology, Zhengzhou University, and Shantou University Medical College, respectively, and participated in previous psychological interviews^9^.

Socioeconomic status (SES) was assessed by subjective socioeconomic status scale (CSSS, Chinese version), a 2-item self-reporting questionnaire with 10 points per item^10^. Item one assesses the patient’s perceived position in the entire social environment, and item two assesses the perceived position in the community, and higher scores relate to the higher SES ladder.

**Propensity score matching (PSM) protocols**

The goal of PSM comparison is to achieve post hoc randomization in a real-world study and the method was applied for chemo and chemoICI group. Propensity scores were calculated with multiple logistic regression and associated variables included all variables as adjusting confounders. In propensity matching, a nearest greedy algorithm was adopted to give head-to-head (1:1) matching between each patient in the two groups. A caliper width of 0.2 was adopted in score matching without replacement. Matching competence was evaluated with the standardized mean difference (SMD), but traditional methods of paired tests were also represented for each baseline variable^11,12^. An SMD > (√ ((n1 +n2)/n1*n2))*1.96 was defined as imbalanced matching (n1 = n2 refers to pre-matched sample sizes)^12^.

**Statistics**

Equivalent statistical tests of difference of score change difference in propensity-score matched samples were performed with Wilcoxon signed-rank tests. Equivalence tests of paired proportion difference, or MOIE/MSIE difference, were performed with McNemar tests. PCI or MOCA score change difference in subgroups with or without incident irAE was compared by linear regression models to adjust for all baseline variables. To calculate the incidence rate, or new case rate, of MOIE/MSIE during follow-up, the Kaplan-Meier survival curve was adopted to estimate the mean event-free survival (EFS) time of comparable groups or subgroups. Paired and independent log-rank tests were adopted to compare the difference in EFS rate. The power remains over 90% to calculate the difference of EFS rate of over 10% difference in both paired and independent log-rank tests, assuming a two-sided, 5% type I error. Multivariate EFS analysis using the proportional hazards model assessed the hazard ratio (95% confidence interval, CI) of irAE that adjusted for all baseline variables, and the continuous variables were not categorized to preserve integrity. Sample sizes were calculated with PASS (version15.0.), and all statistical analysis was performed in R (version 4.0.5) software. Plots of prevalence were drawn with GraphPad Prism (version 8).

**Exclusion or dropout analysis**

Overall, 2218 patients were screened during the recruitment period at the outpatient clinic by the oncologists and the coordinators, but 1557 patients gave consent to the full-length follow-up.

In the 3-month session, 27 dropout patients could not be contacted, and 9 patients dropped out due to careless questionnaire handling. Excluded 30 patients changed their regimen to chemotherapy and targeted therapies due to poor tolerance to diarrhea adverse events (N = 19), fatigue (N =4), or headaches (N = 7). These patients were contacted thereafter and reported mild to moderate cognitive impairment (PCI < 59, N = 18), severe cognitive decline (PCI < 34, N = 2), and unknown results (N = 10).

In the 6-month follow-up session, 32 dropout patients cannot be contacted. 25 patients were contacted and dropout because reported no change of PCI beyond MCID (N = 22) or decreased outside MCID (N = 3). In the chemoICI, 3 patients were excluded because of intolerance (1 patient had a persistent low fever and 2 patients reported pneumonitis). On further reporting by attending oncologists, all 3 patients had no such events. In the chemo group, all 6 excluded patients opted for ICI therapies in other hospitals.

In the 9-month follow-up, MOCA tests were not given due to pandemic-associated lockdown and neurologists or coordinators failed to arrive at the study scene. Only self-reported PCI questionnaires were done. Of the dropouts, 28 patients were contacted and did not wish to continue cognitive assessment, while the other 2 patients had emergencies and could not be contacted. The reasons for the exclusion of 34 patients were 1) change into ICI-based regimens (N = 29) and 2) did not continue treatment (N = 5).

In the 12-month follow-up, 17 patients could not be contacted, and 7 patients were contacted thereafter without further information or data. The reasons for the exclusion of 33 patients were 1) the change of regimen to ICI-based (N = 13) and 2) the change of ICI-based regimen to chemotherapy (N = 20).

| **Supplementary Table1. Immune checkpoint inhibitor types after matching** | | |
| --- | --- | --- |
|  | Number | Percent |
| Dervalumab | 68 | 14.8 |
| Nivolumab | 107 | 23.3 |
| Pembrolizumab | 124 | 27.0 |
| Sintilimab | 84 | 18.3 |
| Toripalimab | 77 | 16.7 |

| **Supplementary Table 2. Risk analysis of incident irAE and outcomes adjusting for baseline variables** | | | | | | | | |
| --- | --- | --- | --- | --- | --- | --- | --- | --- |
| **Variables** | **MOIE** | | | | **MSIE** | | | |
|  | **HR** | **95% CI** | | **p** | **HR** | **95% CI** | | **p** |
| irAE (ref. never) | 2.2 | 1.64 | 2.94 | < 0.01 | 2.11 | 1.59 | 2.8 | < 0.01 |
| Baseline Variables | | | | | | | | |
| Dervalumab (n = 68) | 1.35 | 0.83 | 2.21 | 0.23 | 1.22 | 0.77 | 1.93 | 0.4 |
| Nivolumab (n = 107) | 0.87 | 0.56 | 1.35 | 0.53 | 0.74 | 0.48 | 1.14 | 0.17 |
| Pembrolizumab (n = 124) | 0.87 | 0.55 | 1.35 | 0.53 | 0.75 | 0.49 | 1.15 | 0.18 |
| Sintilimab (n = 84) | 0.83 | 0.51 | 1.36 | 0.47 | 0.87 | 0.55 | 1.36 | 0.53 |
| Toripalimab (n = 77, ref.) |  |  |  |  |  |  |  |  |
| MOCA scores (continuous) | 0.94 | 0.9 | 0.99 | 0.02 | 0.96 | 0.92 | 1.01 | 0.12 |
| ECOG-PS (continuous) | 0.99 | 0.85 | 1.16 | 0.93 | 1.06 | 0.91 | 1.23 | 0.43 |
| Chemotherapy history (ref. Naive) | 1.31 | 0.84 | 2.05 | 0.23 | 0.85 | 0.53 | 1.37 | 0.51 |
| Pt-based chemotherapy (ref. other types) | 1.04 | 0.73 | 1.48 | 0.83 | 1.21 | 0.85 | 1.71 | 0.29 |
| Illiteracy (ref. no) | 1 | 0.73 | 1.38 | 0.99 | 0.93 | 0.67 | 1.28 | 0.66 |
| stage (continuous) | 0.95 | 0.79 | 1.15 | 0.63 | 1.24 | 1.03 | 1.49 | 0.02 |
| NPC | 0.86 | 0.6 | 1.23 | 0.4 | 1.03 | 0.71 | 1.49 | 0.88 |
| NSCLC | 1.12 | 0.79 | 1.57 | 0.53 | 1.36 | 0.98 | 1.89 | 0.06 |
| CRC (ref.) |  |  |  |  |  |  |  |  |
| FACT-G (continuous) | 1.00 | 0.97 | 1.03 | 0.88 | 0.99 | 0.96 | 1.02 | 0.6 |
| SES (continuous) | 0.92 | 0.89 | 0.96 | < 0.01 | 0.96 | 0.92 | 0.99 | 0.02 |
| PCI (continuous) | 1.02 | 1.00 | 1.03 | 0.11 | 1.01 | 0.99 | 1.03 | 0.21 |
| Diabetes (ref. no) | 1.18 | 0.85 | 1.63 | 0.33 | 1.21 | 0.89 | 1.65 | 0.22 |
| Age (continuous) | 1.06 | 1.05 | 1.08 | < 0.01 | 1.02 | 1.01 | 1.03 | 0.01 |
| Sex (ref. female) | 0.98 | 0.72 | 1.35 | 0.92 | 0.96 | 0.71 | 1.3 | 0.82 |
| Opioid (ref. no) | 0.91 | 0.66 | 1.24 | 0.54 | 0.94 | 0.7 | 1.27 | 0.7 |
| BMI (continuous) | 0.99 | 0.95 | 1.04 | 0.71 | 0.99 | 0.95 | 1.04 | 0.71 |
| Analysis was carried out in Cox proportional hazards models. MOIE, meaningful objective impairment event; MSIE, meaningful subjective impairment event; irAE, immune-related adverse events; ECOG-PS, Eastern Cooperative Oncology Group-Performance Score; MOCA, Montreal cognitive assessment; Pt, platinum; NPC, nasopharyngeal carcinoma; NSCLC, non-small cell lung cancer; CRC, colorectal carcinoma; FACT-G, functional assessment of cancer treatment-general; SES, socioeconomic status; PCI, perceived cognitive impairment; BMI, body mass index; HR, hazard ratio; 95%CI, 95% confidence interval. | | | | | | | | |

| Pearson correlation of PCI and MOCA scores after matching | | | |
| --- | --- | --- | --- |
|  | MOCA | | |
|  | MOCA-3 | MOCA-6 | MOCA-12 |
| PCI-3 | 0.37 | NA | NA |
| PCI-6 | NA | 0.42 | NA |
| PCI-12 | NA | NA | 0.26 |

Reference

1 Ma, Y. *et al.* Patient-oncologist alliance and psychosocial well-being in Chinese society strongly affect cancer management adherence with cancer of unknown primary. *Psychooncology* **26**, 991-998, doi:10.1002/pon.4245 (2017).

2 Ma, Y. *et al.* Volume-based predictive biomarkers of sequential FDG-PET/CT for sunitinib in cancer of unknown primary: identification of the best benefited patients. *Eur J Nucl Med Mol Imaging* **44**, 199-205, doi:10.1007/s00259-016-3504-4 (2017).

3 Wagner, L. I., Sweet, J. J., Butt, Z., Lai, J. S. & Cella, D. Measuring patient self-reported cognitive function: Development of the Functional Assessment of Cancer Therapy–Cognitive Function Instrument. *The Journal of Supportive Oncology* **7**, W32-W39 (2009).

4 Wagner, L. I. *et al.* Patient-Reported Cognitive Impairment Among Women With Early Breast Cancer Randomly Assigned to Endocrine Therapy Alone Versus Chemoendocrine Therapy: Results From TAILORx. *Journal of Clinical Oncology* **38**, 1875-1886, doi:10.1200/JCO.19.01866 (2020).

5 Janelsins, M. C. *et al.* Cognitive Complaints in Survivors of Breast Cancer After Chemotherapy Compared With Age-Matched Controls: An Analysis From a Nationwide, Multicenter, Prospective Longitudinal Study. *Journal of Clinical Oncology* **35**, 506-514, doi:10.1200/JCO.2016.68.5826 (2016).

6 Cuzzubbo, S. *et al.* Assessing cognitive function in patients treated with immune checkpoint inhibitors: A feasibility study. *Psychooncology* **27**, 1861-1864, doi:10.1002/pon.4725 (2018).

7 Mills, K. A. *et al.* Cognitive impairment in Parkinson's disease: Associations between subjective and objective cognitive decline in a large longitudinal study. *Parkinsonism Relat Disord* **80**, 127-132, doi:10.1016/j.parkreldis.2020.09.028 (2020).

8 Siciliano, M. *et al.* Correlates of the discrepancy between objective and subjective cognitive functioning in non-demented patients with Parkinson's disease. *J Neurol* **268**, 3444-3455, doi:10.1007/s00415-021-10519-4 (2021).

9 Liu, N. *et al.* Prevalence and predictors of PTSS during COVID-19 outbreak in China hardest-hit areas: Gender differences matter. *Psychiatry Res* **287**, 112921, doi:10.1016/j.psychres.2020.112921 (2020).

10 Goldman, N., Cornman, J. C. & Chang, M. C. Measuring subjective social status: a case study of older Taiwanese. *J Cross Cult Gerontol* **21**, 71-89, doi:10.1007/s10823-006-9020-4 (2006).

11 Ziegel, Eric & R. Statistical Methods for Rates and Proportions (Book). *Technometrics* (2004).

12 Austin, P. C. Balance diagnostics for comparing the distribution of baseline covariates between treatment groups in propensity-score matched samples. *Stat Med* **28**, 3083-3107, doi:10.1002/sim.3697 (2009).
